# Supplementary material for: Absence of toxin gene transfer from Clostridioides difficile strain 630Δerm to nontoxigenic C. difficile strain NTCD-M3r in filter mating experiments
Source: PLoS One. 2022 Jun 29;17(6):e0270119. doi: 10.1371/journal.pone.0270119 (PMC9242483; doi:10.1371/journal.pone.0270119)

Figure 1A

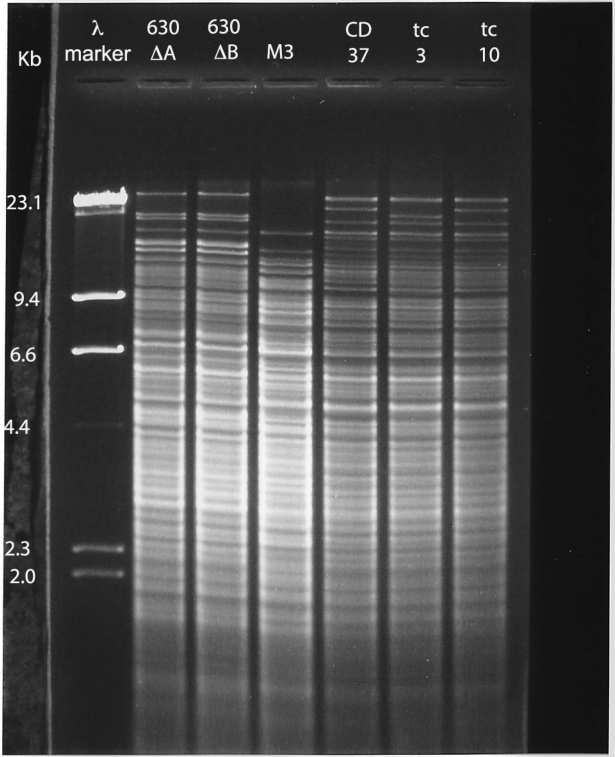

C:\Users\MRL Research Lab\Desktop\New Camera REA gels\20200325ac1.sgd
1585230223 MRL Research Lab 3/26/2020 1:43:43 PM 2.6117 seconds
Dye: Ethidium Bromide - Lights : TLUM - Mid Wave - Filter: UV06

Figure 1B.

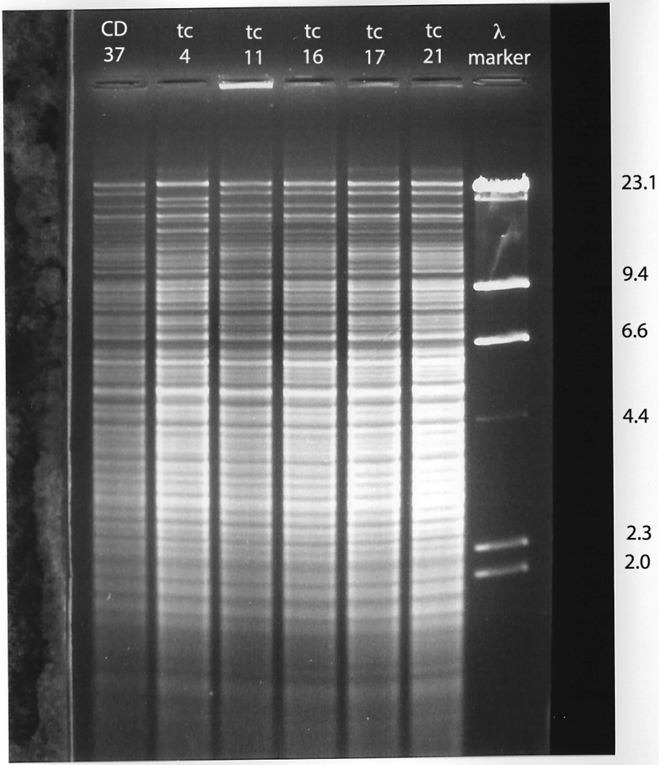

C:\Users\MRL Research Lab\Desktop\New Camera REA gels\20200325ac2.sgd
1585230780 MRL Research Lab 3/26/2020 1:53:00 PM 2.2815 seconds
Dye: Ethidium Bromide - Lights :TLUM - Mid Wave - Filter: UV06

20180517SPSPCRtcdBGel2.sgd
1526587734 MRL Research Lab 5/17/2018 8:08:54 PM 0.6678 seconds
Dye: Ethidium Bromide - Lights :TLUM - Mid Wave - Filter: UV06

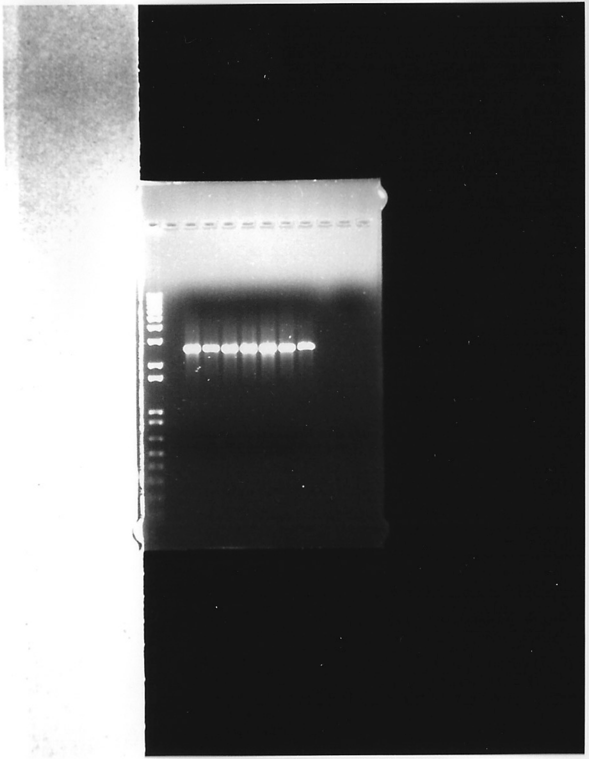

Figure 2A.

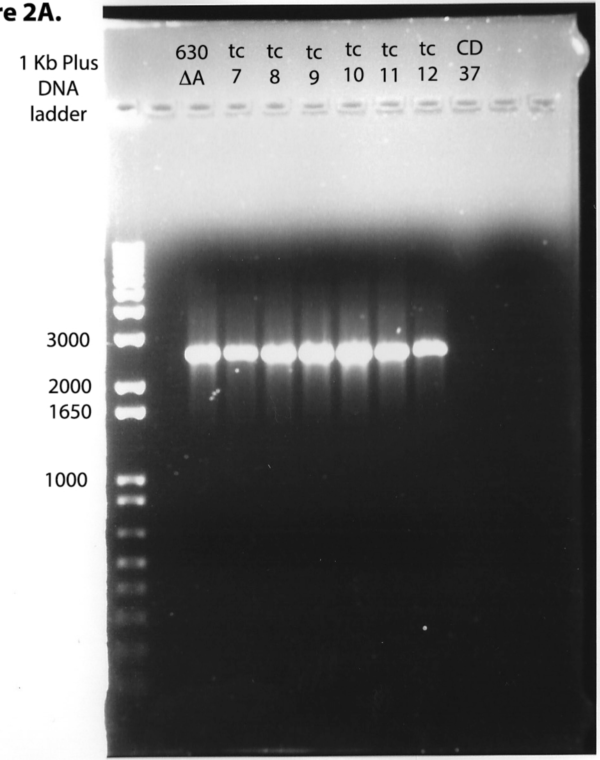

630	tc	tc	tc	tc	tc	CD
ΔA	17	18	19	20	21	37

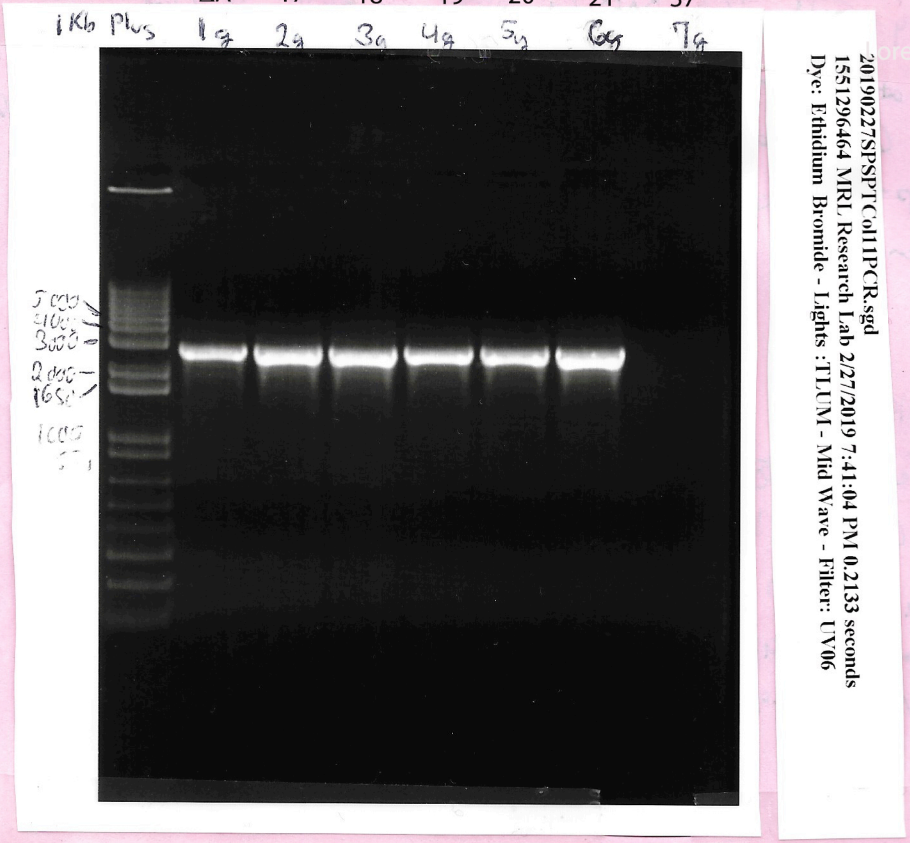

22-04-14SPS Gel Series P edit.sgd
1649955637 MIRL Research Lab 4/14/2022 5:00:37 PM 0.2903 seconds
Dye: Ethidium Bromide - Lights :TLUM - Mid Wave - Filter: UV06

Figure 3.

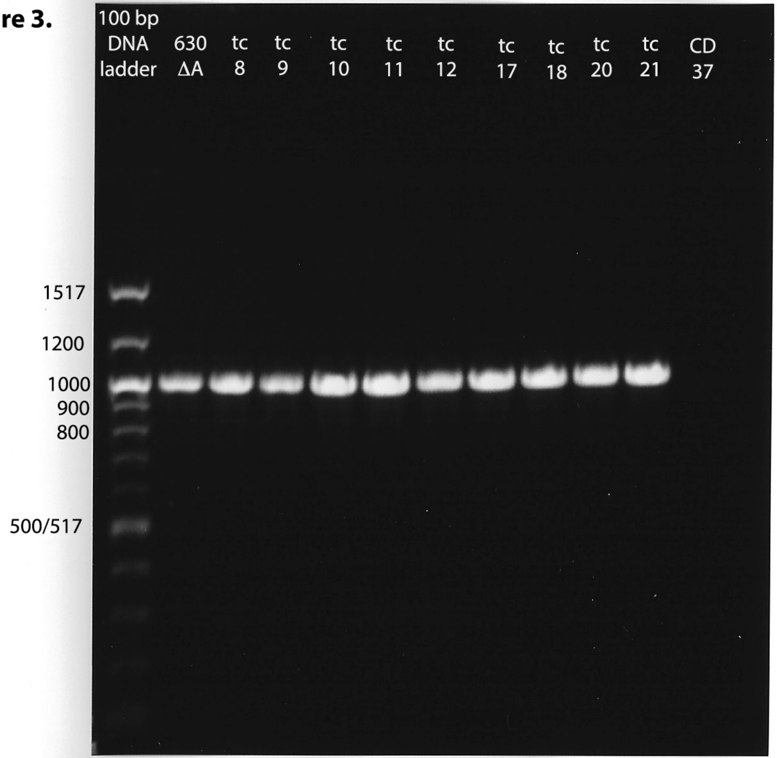

Supplement: S1 Raw images — (PDF) [file pone.0270119.s001.pdf]
